# When parental care hurts: extended parental care and the evolution of overparenting

**DOI:** 10.1093/emph/eoaf027

**Published:** 2025-10-21

**Authors:** David W Lawson, Zhian Chen

**Affiliations:** Department of Anthropology, University of California, Santa Barbara, CA, USA; Department of Anthropology, University of California, Santa Barbara, CA, USA

**Keywords:** parental investment, maladaptation, childcare, child health

## Abstract

In recent years, childrearing in high-income countries has become described as ‘relentless’ in its demands on parents. In response to growing delays in social and financial independence, public health professionals have even advocated for redefining adulthood to begin at later ages. There is also growing concern, not just about the wellbeing of exhausted parents, but of children whose parents are deemed to provide developmentally inappropriate care that may undermine their independence and foster poor mental health. In this review, we describe such ‘overparenting’, an intensive style of modern parenting considered costly to both parent and offspring wellbeing, as a phenomenon of increasing public health relevance, before putting these concerns into an evolutionary framework. We characterize overparenting as an extreme and maladaptive continuation of trends in extended parental care that have characterized much of human (evolutionary) history, highlighting four relevant socioecological shifts hypothesized to incentivize increases in parental care: (i) lowering extrinsic risk, (ii) increased reliance on skill-intensive production, (iii) escalating intergenerational wealth transfers, and (iv) reduced availability of alloparents. From this perspective overparenting within high-income countries presents an underappreciated example of evolutionary mismatch to prevailing socioecological conditions. To conclude, we discuss how an evolutionary perspective on overparenting may help promote new research directions and inform the design of initiatives aimed at improving both parent and child wellbeing.

## INTRODUCTION

In contemporary high-income countries, both popular and public health discourse surrounding parenting has referred to the demands of childrearing as ‘relentless’ [1, 2]. Research from across the social sciences confirms that parental care is skyrocketing. This is evident, for example, in time devoted to childcare activities [3–7]. Compiling data across 11 high-income nations between 1965 and 2012, Dotti Sani and Treas [7] estimate that, despite declining family sizes, among parents living with at least one child under 13 years, the average time mothers spent on childcare activities increased from 54 to 104 minutes daily, while fathers saw a corresponding increase from 16 to 59 minutes. Note that these numbers underestimate total time allocation to childcare by focusing only on relatively intensive self-reported direct care activities, such as reading to children or helping them with homework, excluding more passive supervisory activities​ [8]. Across high-income nations there are also widespread socioeconomic gradients in time allocation, which have typically remained stable or increased in magnitude over time, with relatively well-educated parents allocating the most time to direct childcare [7, 9, 10]. While direct care from fathers has increased over time more so than for mothers, mothers everywhere bear the burden of routine childcare duties, such as feeding and bathing, whereas fathers sometimes engage more in recreational activities, like play [11–13]. These inequalities can lead to harsh trade-offs between reproduction and career progression for women [14, 15].

Parallel trends are evident in financial investments in children. In the USA, for example, the estimated cost of raising a child to age 17 for the average middle-income family reached $233 610 by 2015, representing a 16% increase (inflation-adjusted) from the 1960s [16]. This rise was most pronounced among affluent families who spent approximately seven times more on their children by 2010 compared to lower-income parents [17]. Furthermore, financial support now frequently extends far into adulthood, particularly among wealthy families, with parents investing more into adult offspring than previous generations [18–20]. Today, fewer than half (45%) of young adults aged 18–34 years in the USA self-report that they are financially independent from their parents [21].

Qualitative shifts in modes of parenting and associated ideology are also apparent. Parenting scholars highlight a cultural diffusion of ‘intensive parenting’​ [22, 23]. Intensive parenting is characterized by structuring much of children’s time with enrichment activities designed to develop skills and secure future opportunities. Such behaviors are most pronounced among higher socioeconomic status families, while lower-income parents, despite sharing similar aspirations, face financial and time constraints that limit intensive involvement, such that parenting prioritizes basic needs, but also allows more freedom for independent development [24]. For example, a recent analysis of Chinese families concludes that university-educated mothers not only spend more time engaged in direct childcare compared to those with less education, but that much of this additional time is spent on educational care focused on cognitive enrichment or school performance [25]. Others highlight associated cultural shifts away from ‘control-focused’ disciplinary styles to nurturing approaches that emphasize warmth, engagement, and support, aligning with increasing prioritization of the emotional well-being and autonomy of children [26]. Anxious parents, from all backgrounds, increasingly turn to expert guidance, such as parenting books and professional advice, rather than tradition or intuition [27, 28].

At a global level, Lancy [29] contrasts *gerontocracy* (elders are the most valued members of society) being gradually replaced by *neontocracy* (the needs of children prioritized above all others). Historians of high-income nations have traced the cultural construction of childhood as a ‘sanctuary’ defined by nurturing and education, with both parents and the state deemed increasingly responsible for sheltering children from the realities of adulthood [30–32]. Accordingly, a rigid boundary between childhood innocence and adult responsibility has become a prominent feature of international human rights activism imposing a universal threshold of adulthood at 18 years [33], including global commitments to ending ‘child marriage’ [34]. Contemporary policy debates demonstrate an appetite for further extending the age threshold of adulthood. Recently, Sawyer *et al.* [35] proposed that public health professionals redefine ‘adolescence’ from 10–19 to 10–24 years, reflecting delayed timings in economic independence, marriage and parenthood, and changing notions of adult maturity.

One might assume that these trends in ‘modern parenting’ (used here broadly to refer to the continuing extension and intensification of parental care within contemporary high-income nations described above) are beneficial for children. However, an emerging literature counters that, for at least a subset of families, parenting has become, not only very costly to increasingly exhausted parents, but detrimental to child wellbeing. In this review, we characterize such concerns about ‘overparenting’ as a phenomenon of growing public health relevance, before placing it into a broader evolutionary framework. Here, we argue that overparenting presents an underappreciated example of evolutionary mismatch to prevailing socioecological conditions. Our review concludes by suggesting future research directions into overparenting informed by an evolutionary perspective on parental care.

## OVERPARENTING AS A PHENOMENON OF GROWING PUBLIC HEALTH RELEVANCE

### What is overparenting?

Recent years have witnessed increased public and academic recognition of overparenting. Overparenting can be considered a subcategory of ‘modern parenting’, characterized by both high levels of care and a developmental misalignment to children’s needs, leading to a potential for harm. It is typified by well-intentioned but controlling and intrusive behaviors such as frequently intervening in conflicts, managing tasks, and shielding children from challenges they could more appropriately handle alone (synonymous terms include ‘helicopter parenting’ or ‘lawnmower parenting’) [36–40]. While there is some inherent subjectivity to this characterization, overparenting can be usefully contrasted with alternative parenting styles. For instance, while overparenting shares characteristics with ‘intensive parenting’ (see above), it is distinguished by the excessive nature of parental regulation and its mismatch with the child’s developmental stage [38, 40]. Related but distinct terms in the literature also include ‘permissive parenting’, which is characterized by high responsiveness but minimal control, while overparenting pairs attentiveness with strict oversight, closely managing children’s experiences [41]. Likewise, ‘authoritarian parenting’ is rooted in a desire to enforce discipline and obedience, while overparenting reflects aspirations to protect children and maximize their chance of success, merging excessive control with deep parental involvement, ultimately hindering a child’s ability to become self-sufficient [36].

Although there is no definitive diagnostic measure of overparenting, several behaviors across childhood into adulthood are typical of the phenomenon. In early childhood, this includes excessive regulation of everyday activities, frequent intervention in problem-solving, overmonitoring, and unnecessary risk avoidance [38, 40]. These behaviors signal to children that they are not competent, reducing opportunities for learning through experience. As children mature into adolescence, overparenting often persists as intrusive involvement in organizing routines, handling peer conflicts, or managing academics [41–43]. There may also be excessive scheduling, boundary-setting, and privacy intrusions, often framed as protective or achievement-driven [42]. Such behaviors are argued to disrupt normative processes of autonomy development and identity exploration. During emerging adulthood, when autonomy is developmentally expected, overparenting can manifest through interference in academic, social, and occupational matters, such as contacting professors or employers or managing adult children’s schedules, along with continued intervention in peer conflict and daily decisions [38, 43]. These examples distinguish overparenting from more general intensive parenting practices that may be costly for parents but typically either beneficial or benign for offspring.

Most research on the topic views overparenting as rooted in self-perpetuating cycles of parental anxiety; whereby overparenting limits child independence, which in turn heightens child anxiety and reinforces a parental tendency to intervene​​ [41, 44]. In general, overparenting tendencies appear most prevalent among relatively affluent families who combine high standards for child achievement [45] with anxiety over child safety and development [22]. Mothers appear more likely than fathers to engage in overparenting, in line with cultural norms emphasizing their primary caregiver role [46–49].

### When parental care hurts

While findings are somewhat mixed across the literature at large, multiple recent meta-analyses and systematic reviews conclude that overparenting behaviors are associated with negative wellbeing outcomes for offspring [37, 42, 50–53]. Overparenting, for instance, has been linked with obsessive perfectionism and an intense fear of negative evaluation, making children prone to self-doubt [54, 55]. During adolescence, it is associated with dependency on parents for decision-making, and in transitions into college life, challenges with social adjustment and reduced academic motivation [56, 57]. In these situations, students prioritize grades over intellectual engagement, while experiencing heightened fears of failure [58]. Overparenting has also been linked to school burnout and risky behaviours, including internet addiction and misuse of pain medications​​ [59–61]. Several studies also suggest overparenting discourages young adults from pursuing ambitious careers, leading to challenges in career exploration and the abandonment of personal goals​ [62]. In studies assessing participants at multiple stages of adulthood it has been linked with higher anxiety and depression, lower self-efficacy and weakened social, academic, and romantic competence [37, 42, 43, 50, 63].

Many of these studies are published within the last decade. As interest grows, we can expect the literature to reach more refined conclusions as methods conform to shared and validated metrics, potential mediators and confounders are more thoroughly investigated, and contingencies depending on socioecological, parental and child characteristics are more fully considered. In this vein, it is interesting that comparative work suggests the negative impacts of overparenting are more severe in the USA than China, where overparenting behaviours have been argued to align more with cultural norms of interdependence [64]. There is also some work suggesting that parent gender is impactful. For example, in one study paternal overparenting was more strongly correlated with lower college student adjustment than was maternal overparenting [65]. In contrast, Van Ingen *et al.* [66] report that maternal overparenting behaviors were more strongly associated with offspring’s lower self-efficacy. However, in a recent meta-analysis by McCoy et al [52] overparenting behaviors were linked with poor adult functions regardless of gender.

## AN EVOLUTIONARY PERSPECTIVE ON EXTENDED PARENTAL CARE

### A gap in the literature

To our knowledge, the now burgeoning social science literature on overparenting has not yet considered the potential relevance of an evolutionary perspective, despite parental care being a key topic of study, particularly within behavioral ecology frameworks [67, 68]. Likewise, while evolutionary social scientists have debated whether modern patterns of childrearing are adaptive or maladaptive (see Section: Overparenting as a product of evolutionary mismatch), we are aware of no work in this tradition that has engaged with the possibility that a subcategory of modern parenting behaviors may actually be costly to offspring wellbeing and fitness. With the objective of promoting synthesis between these disparate literatures, in this section we introduce an evolutionary perspective on variability in human parenting and the socioecological factors that favor extended parental care. From this foundation, we then characterize overparenting as a product of evolutionary mismatch in the following section.

### Human parental care in comparative perspective

‘Parental care’ is classically defined in the evolutionary sciences as any behaviour enhancing offspring fitness that originated and/or is maintained by selection for that function, without necessarily being costly to the parent [69, 70]. Recognizing overparenting as costly to offspring requires that we amend this definition to include behavioural analogues that were ancestrally, but not presently, beneficial to offspring. Note ‘parental investment’ refers more narrowly to care that is demonstrably costly to a parent’s ability to invest in other components of fitness [70, 71], and so is subject to life history trade-offs, and conflicts of interest between family members (e.g. sibling or parent-offspring conflict). Accordingly, we reserve parental investment for behaviour where costs are substantiated.

As mammals, humans inherit internal gestation and lactation, establishing a high minimum maternal investment. However, unlike most other primates, paternal care is relatively high, albeit context-dependent [72, 73]. Humans are also unusual among apes in the extent to which they recruit alloparents [74], and to which they ‘stack offspring’, with weaned children remaining dependent and different forms of care supplied simultaneously to offspring at distinct ages [75]. Moreover, we exaggerate trends towards extended parental care observed among primates in comparison to other mammals. Our offspring are highly altricial and, while weaned relatively early compared to other apes, are rarely independent until well into the second or third decade [76]. Human parental care is also extraordinarily plastic, varying in form and duration across and within societies (Table 1). Context-dependent parental care has been documented in many species [77], but given the exceptional socioecological range of humans, and our unique capacities for social learning, the flexibility of human parenting is arguably unparalleled.

**Table 1:** Parental care in humans: Form, socioecological variation and scope for sibling competition.

Form of parental care	Socioecological variation	Scope for sibling competition	Exemplary reference(s)
Gestation	Minimal	High	[78]
Breastfeeding	Children are typically weaned earlier in high-income countries.	High	[79, 137]
Provisioning (i.e. foraging, hunting, food preparation)	Variation in maternal and paternal roles depending on subsistence strategy.	High, but reduced when children are active producers.	[97, 138]
Protection, shelter	Variation in physical and social threats, some may be extrinsic risks.	Low	[139]
Health care (e.g. washing, taking to the doctor, etc.)	Marked variation in access to health services and in local health risks.	High if payments required, less so if time-based and or if pathogen risk is extrinsic.	[94, 140]
Emotional Closeness / Affection / Attention	Infant-caregiver physical contact greater among small-scale societies. Affection and attention exaggerated among modern high-income countries.	Modest	[141, 142]
Informal teaching, including socialization	Mode and extent of teaching varies, as does the importance of skill accumulation in subsistence.	Modest	[83, 84]
Schooling	Primary schooling is now almost universal, secondary and tertiary education less so.	High, especially if school fees or opportunity costs are present.	[88, 143]
Marriage payments	Often substantial among pastoralists and agriculturalists. Some practice bridewealth, others dowry.	High, sex specific	[84, 102, 103]
Wealth transfers and inheritance	Low in subsistence foragers, but otherwise often substantial. Some practice patrilineal inheritance, others matrilineal inheritance	High, sex specific.	[85, 88, 89, 144]
Child rearing assistance	Proximity to grandparents depends on residence system, maternal grandparents usually more important.	High for practical support, low for emotional support.	[145–147]

Adapted from [67]

Parental care has energetic, interpersonal and material dimensions [67]. Energetic care requires direct caloric expenditure, including gestation and breastfeeding [78, 79]. Sibling competition over energetic resources is intense [79, 80]. Excluding rare cases of surrogacy, gestation is carried out by mothers, and while allomaternal nursing is not uncommon cross-culturally, mothers typically do the large majority of breastfeeding [81]. Interpersonal care usually requires direct parent-offspring interaction and includes behaviours associated with attention, affection and encouragement, and the transfer of skills and knowledge via teaching and socialization [82, 83]. There is evidence, albeit primarily from high-income countries, of sibling competition over interpersonal investments, e.g. children in larger families spend less time with parents [13]. Finally, material care encompasses provisioning, providing shelter, and investments in health care and formal schooling. Resource transfers can also be critical in obtaining marriage partners via bridewealth or dowry [84] and pivotal in establishing independence as offspring disperse or at inheritance following parental death [85]. Wealth transfer via these channels leads to heritable inequalities [86], with the division of capital a key site of sibling conflict [87–89]. All these forms of care have been studied extensively by evolutionary social scientists (Table 1).

### Socioecological drivers of extended parental care in humans

**Figure 1:**
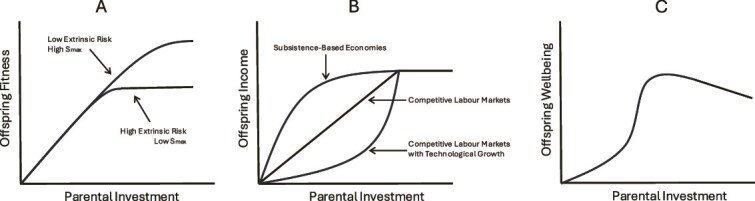
Hypothesized relationships between parental investment and offspring outcomes. A) Early studies in evolutionary anthropology, focused on subsistence-based economies, emphasized the role of extrinsic risk in determining optimal allocations of parental investment. Offspring fitness increases with additional parental investment until a saturation point is reached (*Smax*), after which further allocations of parental investment do not improve offspring fitness. In contexts of relatively low extrinsic mortality *Smax* is reached later favoring extended parental investment and corresponding lower fertility (adapted from [92], see also [91]); B) Kaplan [100] proposed that transitions from subsistence-based economies to competitive skill-intensive labor markets radically alter the relationship between parental investment and offspring income, such that marginal returns to parental investment escalate at higher levels. Our evolved psychology responses maladaptively to this change, leading to exceptionally high parental investment per offspring and corresponding low fertility (adapted from [100]); C) There is now mounting evidence (see text) that the recent emergence of highly intensive parenting practices within high-income populations are not just maladaptive for parental fitness, but directly costly to offspring wellbeing. From this perspective, increasing parental investment benefits offspring wellbeing, but after a certain point increasing investment further is detrimental. Whether or not ‘overparenting’ is costly for offspring fitness remains to be investigated.

Hereafter, we use ‘extended parental care’ to refer to situations wherein the duration and/or intensity of parental care has increased over time, including trends in modern parenting described at the opening section of this paper. Broadly speaking, four main socioecological drivers are understood to favor extended parental care in humans. In each case, we can consider species-level variation, comparing chimpanzees (a proxy for our shared ancestry) to human foragers, along with plasticity across our socioecological range brought about by subsistence transitions (i.e. to farming, pastoralism and market-integrated economies). Here, we introduce theoretical concepts that situate an understanding of extended parental care (see also Fig. 1), which may be useful in understanding the evolution of overparenting.

#### Lowering extrinsic risk

Less than 50% of offspring survive to adulthood among extant foragers [90]. While high by contemporary global standards, this represents low mortality compared to chimpanzees, indicating benefits to our new foraging niche. When early life mortality is high, it is typically relatively extrinsic (i.e. survival depends mostly on factors outside of parental control, such as vulnerability to natural disasters, intergroup conflict or high pathogen loads), rather than ‘intrinsic’ i.e. preventable by increased parental investment [80, 91, 92]. As such, mortality reduction across human evolution likely reinforced payoffs to extended parental care by reducing the need for bet hedging i.e. spreading investments across multiple offspring to offset unpredictable mortality. In contrast, transitions from foraging to farming appear to, on average, increase child mortality following elevated pathogen transmission as population density increases [93]. With market integration, extrinsic mortality declines in tandem with improvements in public health services, increasing reliability in the returns to extended parental care. Direct evidence to support the impact of extrinsic risk on parenting is limited [91], however selective neglect of sickly children has been observed in high mortality contexts, freeing resources to channel into alternative kin whose survival is more likely [94–96].

#### Increased reliance on skill-intensive production

Our elongated juvenile dependency coevolved with movement into a novel foraging niche, with increased time required to acquire the necessary skills to hunt successfully and produce sufficient calories to first feed oneself and later support junior family members [97]. From this perspective, extending childhood is a strategy to make more productive adults. With hunting taking a long time to become proficient, children may do little to offset their costs to parents [97]. In contrast, in transitions to farming or pastoralism, children are often enrolled in less skill-intensive tasks, underwriting costs to parents and freeing resources for investment in alternative fitness functions [98]. When children are active producers, some aspects of parental care (i.e. provisioning in early life) can be reduced. However, with market integration, children’s work is limited with time increasingly allocated to schooling. While some children appear able to combine productive work and school [99], schooling is also typically financially costly, especially when continued into higher education. In the face of increasingly skill-intensive labor markets, a continuing expansion of educational investment is favored by a corresponding escalation of returns to elongated dependency and parental investment in offspring status [100].

#### Escalating intergenerational wealth transfers

Extrasomatic wealth (e.g. land, cattle or cash) is absent in chimpanzees and limited among foragers [97]. Accordingly, large sibships appear beneficial in adulthood among foragers, due to a relative lack of resource competition and ample opportunities for nepotistic affiliation [101]. In contrast, wealth transfers in pastoralists and agriculturalists amplify the reach of parental care [86]. Consequently, sibling competition, especially among the inheriting sex (usually men), may reduce individual reproductive success [87, 102–104]. Modernization is accompanied by increases in wealth, further extending the scope of intergenerational transfers and so reach of parental care. The transmission of wealth across generations is highly evident in multigenerational studies of high-income nations [88]. Yet, to varying degrees, welfare states also buffer risks of falling into poverty, countering the inheritance of inequality [86].

#### Reduced availability of alloparents

Alloparental help is minimal among chimpanzees [105]. Juveniles, for example, do not provision their younger siblings [106]. In contrast, human alloparents, particularly grandparents and elder siblings, lower the burden of care, explaining why we are capable of relatively tight birth intervals [107]. Alloparenting by kin is ubiquitous in small-scale societies, albeit with considerable variation in which kin help [107–109]. Market integration brings a fragmentation of kin ties and nuclearization of the family as mobility increases, intergenerational living arrangements decline and fertility falls [110–112]. Parents still rely on alloparents [113] but increasingly turn to paid childcare. When such care is unavailable, either due to lack of resources to pay for assistance, or when access is limited (e.g. due to pandemic lockdowns), modern parenting places considerable pressure on parents balancing careers and parenthood [114].

## OVERPARENTING AS A PRODUCT OF EVOLUTIONARY MISMATCH

### Modern parenting behaviors are maladaptive for parents

The preceding section identifies a suite of socioecological drivers that incentivize extended parental care across and within species. For the most part, such extensions in parental care are likely adaptive responses to prevailing conditions. However, modern parenting trends present an extreme extension of parental care that ostensibly defies adaptive decision-making. Following influential theoretical work by Kaplan *et al.* [100], evolutionary social scientists now largely agree that modern parenting is maladaptive because it has coevolved with remarkably low fertility rates. According to this account, our reproductive psychology evolved in ancestral socioecologies where (i) children were relatively cheap to raise, (ii) returns to investment in offspring ‘embodied capital’ (i.e. education, skills, training) were relatively low, and (iii) childhood mortality was relatively high. Modern societies invert these conditions: kids are extremely costly in time and money, advanced schooling is rewarded in the labor market, and almost all children survive. The result is an evolutionary mismatch. Our evolved capacity to regulate parental care in response to predicted pay-offs collides with a new environment that makes each additional child far more expensive, favoring exceptionally low fertility that is no longer fitness-maximizing. Complementing Kaplan *et al.*’s work [100], while remaining more open-minded about the ultimate fitness consequences of observed trends, Mace [115, 116] has further likened modern parenting to a runaway process, in which ever escalating levels of parental investment drive fertility lower as we attempt to live up to shifting standards of social competition, even as tangible embodied capital benefits of continued investment become questionable.

Several mathematical models have outlined hypothetical scenarios wherein a coevolution of modern low fertility and extended parental care could be still considered adaptive in the long term, provided sufficient advantages are gained by offspring which in turn enhance their reproductive success [117–119]. However, these models have been dismissed because they are sharply contradicted by the available empirical evidence. All else equal, being raised with fewer siblings leads to relatively superior health [120] and enhanced socioeconomic status [89, 121], but such wellbeing outcomes are ultimately not very influential to eventual reproductive success. Multigenerational studies of multiple high-income populations demonstrate that having more children on average consistently leads to more long-term descendants [88, 121–124]. As such, we can conclusively state that modern parenting trends, to the extent to which they coevolve with low fertility, are best considered maladaptive at the parental level.

### Overparenting is likely costly for offspring fitness

While likely maladaptive for parents, evolutionary social scientists have generally assumed extended parental care is always beneficial for offspring (i.e. via greater parental investment and subsequent relative improvements in wellbeing). The key distinction of overparenting is that it appears to cause harm to offspring (see Section: Overparenting as a phenomenon of growing public health relevance). As we have outlined above, compared to chimpanzees, human childhood and adolescence are characterized by extended dependency, which evolved to support flexible skill acquisition and adaptive behavioural development [97]. These life stages did not evolve to be micromanaged, but rather healthy development anticipates graduated exposure to challenge and failure, ultimately fostering resilience, decision-making capacity and self-efficacy. In this sense, from an evolutionary perspective, overparenting can be viewed as a developmentally misaligned strategy that interrupts evolved pathways of growth, learning and adaptive function. As such, we suggest that overparenting is best considered a subcategory of modern parenting that is not just costly for parental fitness, but it is likely costly for offspring fitness as well. If this is true, overparenting should be predictive of relatively low reproductive success, which may transpire via difficulties gaining or retaining a partner and/or reductions in fertility within a relationship. Few existing studies directly address these possibilities. However, consistent with our prediction, overparenting has been linked to difficulties with emotional intimacy in adulthood, stifling relationship formation [125, 126]. Furthermore, it has been associated with uncertainty over adult children’s own parenting abilities associated with an undermined confidence in independent decision-making​​ [127], which could logically reduce motivation for continued childbearing.

Existing evolutionary research on harmful parenting practices has concentrated on underinvestment or withdrawal of care, including selective neglect, abandonment or even infanticide, often interpreted as adaptive responses to harsh trade-offs when offspring survival prospects are low [67]. Overparenting presents a novel counterpart to these phenomena. Instead of harm arising from too little care, it results from excessive and developmentally inappropriate care and is seemingly motivated by parental efforts for offspring to thrive rather than fail. From this perspective, overparenting extends the evolutionary literature on harmful parenting by demonstrating that misalignment at either extreme of the parental care spectrum can be costly for offspring wellbeing, and ultimately fitness.

## CONCLUSION AND FUTURE RESEARCH DIRECTIONS

The ‘evolutionary demography’ of fertility behavior is now a burgeoning field defined by theoretical and methodological exchange with mainstream demography [128]. However, equivalent exchange between non-evolutionary and evolutionary social scientists studying parental care is lacking. To this end, the objective of our review has been to connect a growing public health orientated literature on both ‘modern parenting’ and ‘overparenting’ to relevant evolutionary scholarship. Fostering greater exchange between these bodies of literature draws attention to several priorities for future research, which in turn may inform the design and evaluation of interventions aiming at improving both parent and child wellbeing. The contemporary relevance of these issues is underscored by a recent advisory statement by the US Surgeon General on the public health impact of parenting stress and its impacts on children [2].

First, our review draws attention to the need for improved measures of parental care and its consequences. Evolutionary research on human parental care has been critiqued for often bypassing the direct measurement of care activity and instead making assumptions about parental care from data on child outcomes [67]. For example, in studies estimating the hypothesized life history trade-off between offspring quantity and ‘quality’ it is typically assumed that differences in parental care mediate observed relationships but care itself is rarely measured [129]. One consequence of this limitation is that we have only a limited understanding of the extent to which alternative forms of parenting behavior (Table 1) meet Trivers’ definition of parental investment; that is, truly costly to parents and beneficial to offspring. Where parental care is measured, focus has tended to be on quantifiable metrics which vary on continuous scales, such as time or resources allocated to offspring. Such measures are highly amenable to statistical analysis and scientific hypothesis testing. In contrast, overparenting is defined relatively subjectively as a qualitatively distinct mode of excessive parental involvement and by its potential to cause harm. Evolutionary social scientists must therefore engage with a broader range of measures if they are to offer value to our understanding of overparenting and its consequences.

Second, the broad comparative framework of evolutionary social science has the potential to stimulate new lines of research into modern parenting and overparenting alike. For example, while we have outlined that (i) reduced extrinsic risks, (ii) increased dependency on skill-intensive production, (iii) increased importance of inherited wealth, and (iv) reduced alloparental support are all relevant socioecological drivers that help to explain the continuing skyrocketing of modern parental care, their precise impact on overparenting behaviors remains to be investigated. While overparenting is most common among high socioeconomic status parents within high-income nations, we are not aware of work trying to distinguish, for example, relative roles of concerns about offspring skill development vs. anxieties over limited alloparental support or wealth inheritance as potential determinants. There is also untapped potential to consider how overparenting may relate to not only to delayed behavioral maturity of offspring, but relatively ‘accelerated’ physiological maturity. The living conditions of contemporary high-income societies are associated with earlier menarche compared to historical records [130], leading to public health concern over, for example, the early sexualization of youth. An evolutionary perspective may be valuable in helping to disentangle the distinct but interacting determinants shaping physiological and behavioral maturity, and future work should consider whether concerns over early puberty amplify parental anxiety about protecting children from risk, further encouraging overparenting.

Finally, at the proximate level, the expertise of both evolutionary psychologists and cultural evolution scholars may be impactful to overparenting research and policy. Evolutionary psychologists have made important strides in understanding the evolved decisions rules and motivations that guide mate selection [131], and there are signs of a growing interest in the psychology of reproductive strategies [132]. However, dedicated studies of parenting attitudes and behavior remain rare. Even without considering potential harms of overparenting, there is mixed evidence that time-intensive parenting benefits children [133]. It is likely that parents respond to perceived rather than actual benefits of childcare, and factors beyond the returns to care when deciding how to parent. Evolutionary thinking may help to guide hypotheses about what motivates care, and alternative modes of involvement, in the absence of direct benefits to offspring, such as for example, as a means of promoting parental attractiveness or signaling commitment to a current or prospective partner [67]. With overparenting entangled with culturally-transmitted ideologies of desirable parenting and growing concerns over ‘keeping up with the Joneses’ [116], future work should take care to incorporate the study of socialization and evolved social learning strategies [134], such as desire to seek conformity and the emulation of prestige in the development of parenting concerns and ideals. Moreover, a cultural evolution perspective may be helpful in untangling the determinants of overparenting, not merely as individual behavior, but as the outcome of intersecting structural, normative and institutional pressures.

Determining the extent to which parents are responsive to information about the amount and/or form of parental care that benefits (or harms) offspring may be particularly informative for policy design. In this vein, Biroli *et al.* [135], recently reported that less-educated British parents perceive the returns to investing in child health to be lower, and that this perception is predictive of both lower health related investments and poorer child health outcomes. There is also evidence that at least some parents are turning against overparenting behaviors in light of information on its harmful consequences. For example, growing awareness of the dangers of overparenting appears to be producing a corresponding backlash movement and production of supportive media favoring less intensive parenting styles [136]. Such observations underline that while evolutionary mismatch is an important theoretical cornerstone of the evolutionary approach to public health, we must not be naive to our capacity for flexible and responsive decision-making. Adaptive lag may account for current patterns, but only time will tell if behavioural trends will shift with growing recognition that, when it comes to parental care, less may sometimes be more.
